# Plants originating from more extreme biomes have improved leaf thermoregulation

**DOI:** 10.1093/aob/mcaf080

**Published:** 2025-05-04

**Authors:** Pieter A Arnold, Monique J White, Alicia M Cook, Andy Leigh, Verónica F Briceño, Adrienne B Nicotra

**Affiliations:** Division of Ecology and Evolution, Research School of Biology, The Australian National University, Canberra, ACT 2600, Australia; Division of Ecology and Evolution, Research School of Biology, The Australian National University, Canberra, ACT 2600, Australia; University of Technology Sydney, School of Life Sciences, Sydney, NSW 2007, Australia; University of Technology Sydney, School of Life Sciences, Sydney, NSW 2007, Australia; Division of Ecology and Evolution, Research School of Biology, The Australian National University, Canberra, ACT 2600, Australia; Division of Ecology and Evolution, Research School of Biology, The Australian National University, Canberra, ACT 2600, Australia

**Keywords:** Alpine, climate warming, desert, heatwave, leaf temperature, limited homeothermy, stomatal conductance, temperate, thermal coupling, thermal offset, thermal sensitivity, thermoregulation

## Abstract

**Background and Aims:**

Many plants have some capacity for leaf thermoregulation via stomatal conductance (*g*_sw_), such that leaf temperature (*T*_leaf_) is rarely coupled with air temperature (*T*_air_). The difference between leaf and air temperature (thermal offset, Δ*T*) and the slope (thermal coupling strength, *β*) is mediated by interactions between the immediate environment of the plant and its leaf traits. The aim of this study was to determine whether species originating from biomes with contrasting environmental conditions (alpine, desert and coastal temperate) would differ in their tendency to thermoregulate in a common environment.

**Methods:**

Using benign-temperature (25 °C) and high-temperature (38 °C) glasshouse treatments, we measured paired canopy *T*_air_ and *T*_leaf_ for 15 diverse species, 5 from each biome, in a common garden experiment. Instantaneous stomatal conductance and a suite of leaf traits were measured and calculated to test for associations with leaf thermoregulation.

**Key Results:**

We found clear evidence for greater leaf cooling occurring during high-temperature exposure, especially in alpine and desert species. The leaves of temperate species were largely warmer than air in both treatments. Thicker leaves with higher water content and high stomatal conductance clearly were more effective at cooling. Species originating from different biomes displayed divergent responses of thermal offset and thermal coupling with leaf traits.

**Conclusions:**

Our findings suggest that plants originating from more extreme biomes have innately greater scope for thermoregulation, especially desert plants, which could better counter the risk of reaching excess temperatures at the cost of higher water loss. Leaf thermoregulation is a complex plant–environment interaction, and our work contributes to the development of more accurate predictions of leaf temperature during heat exposure across diverse species and biomes.

## INTRODUCTION

Extreme climatic events are major contemporary challenges to terrestrial plants (Perkins-Kirkpatrick *et al.*, 2024). Pulse events that include periods of extremely high temperatures, such as heatwaves, are increasing in frequency, intensity and duration in Australia and are expected to worsen in future decades (Cowan *et al.*, 2014; Perkins-Kirkpatrick and Lewis, 2020). Against the backdrop of accelerated climate warming, heat pulses will expose plants to acute high temperatures that far exceed their typical range (Harris *et al.*, 2018). High temperature affects many physiological and biochemical processes in plants, potentially inflicting injury to tissues and membranes that maintain homeostasis (Goraya *et al.*, 2017). Plants have therefore developed an arsenal of mechanisms to help avoid, tolerate or acclimatize to high temperature to reduce the impact of heat on plant function (Goraya *et al.*, 2017; Nievola *et al.*, 2017; Deva *et al.*, 2020; Geange *et al.*, 2021).

Leaf temperature (*T*_leaf_) is central to the maintenance of photosynthetic performance and metabolic homeostasis (Gates, 1968; Jones, 2014). It is now well established that plants are not necessarily poikilotherms that conform to air temperatures (*T*_air_) of their environment (Mahan and Upchurch, 1988; Michaletz *et al.*, 2015), which is apparent from individual leaves (Tserej and Feeley, 2021) to ecosystem canopies (Guo *et al.*, 2023). *T*_leaf_ can decouple markedly from *T*_air_ in a range of environmental conditions but is typically exacerbated during periods with high sun exposure and low wind and during heat pulses and heatwaves (Leigh *et al.*, 2012, 2017; Hüve *et al.*, 2019; Slot *et al.*, 2021; Kitudom *et al.*, 2022; Kullberg *et al.*, 2023; Manzi *et al.*, 2024). Leaves are often warmer than air when *T*_air_ is cold and there is sufficient insolation, whereas leaves can be cooler than air when *T*_air_ is warm and water is available to the plant for transpiration (Michaletz *et al.*, 2015), i.e. plants can exhibit limited homeothermy.

The limited homeothermy hypothesis posits that plants can maintain an operative temperature by reducing *T*_leaf_ through active transpiration (Mahan and Upchurch, 1988). Decoupling of *T*_leaf_ from *T*_air_ occurs owing to structural properties of the leaf and thermoregulatory behaviour (Michaletz *et al.*, 2015; Lin *et al.*, 2017; Tserej and Feeley, 2021). Mechanisms of thermoregulation in plants can be described simply as either passive or active via structural and physiological means (Drake, 2023). Intrinsic leaf structural traits allow plants to thermoregulate passively (e.g. leaf lamina area or width; Leigh *et al.*, 2017) and avoid rapid excursions to temperature extremes by slowing heat transfer (e.g. leaf thickness and water content; Vogel, 2009; Leigh *et al.*, 2012). Differences in leaf structural traits are driven by differences in biomes or environmental conditions (Gibson, 1998; Lusk *et al.*, 2018); in a common environment, leaf trait differences might be less pronounced among species (Reich *et al.*, 2003). In contrast to passive influences of leaf structural traits, plants can thermoregulate actively by dynamically adjusting stomatal conductance of water vapour (hereafter, *g*_sw_) (Michaletz *et al.*, 2015; Guo *et al.*, 2022). Stomata can be regulated finely between closed and fully open states to optimize gas exchange and water loss and to regulate *T*_leaf_ in the absence of photosynthesis (Gates, 1968; Matthews *et al.*, 2017; Drake *et al.*, 2018).

During drought stress, species differ in their stomatal behaviour and water-management strategies along a continuum from avoidance (stomatal closure to limit water loss) to tolerance (stomatal opening, which allows dehydration), sometimes referred to as isohydric and anisohydric (Klein, 2014; but see Hochberg *et al.*, 2018). In high-temperature conditions, regulation of *T*_leaf_ depends strongly on active evaporative cooling via transpiration (Drake *et al.*, 2018; Marchin *et al.*, 2022). During a record heatwave that exceeded 48 °C, Posch *et al.* (2024) found dynamic patterns of *T*_leaf_ during a common garden experiment. *T*_leaf_ was typically lower than *T*_air_ when water was readily available, which enabled *g*_sw_ to be relatively high. Thereafter, a water-stress treatment applied during extreme heat led to *T*_leaf_ exceeding *T*_air_ (disrupting homeothermy) when water availability was low and *g*_sw_ was near zero (Posch *et al.*, 2024). If high temperatures coincide with water limitation, many plant species are unable to transpire to dissipate heat, hence leaves can reach damaging temperatures (Cook *et al.*, 2021; Marchin *et al.*, 2022; Posch *et al.*, 2024). In contrast, other species have recently been observed to maintain partly open stomata in high temperatures, even in droughted plants (Marchin *et al.*, 2022).

There is a clear trade-off between water use and active thermoregulation (Fauset *et al.*, 2018). However, species that originate from distinct biomes and/or that have different leaf traits will differ in their thresholds for when and how much stomata are opened based on their relative position on the avoidance–tolerance spectrum (Marchin *et al.*, 2022). Leaf thermoregulation therefore involves more than the biophysical effects of structural leaf traits; stomatal strategy makes a substantive difference to leaf temperature. We therefore expect that species originating from contrasting environments would have developed divergent leaf thermoregulation tendencies or different thermal coupling responses (Blonder and Michaletz, 2018). Cooling via stomatal behaviour can be more effective than the mediating effects of passive leaf traits when sufficient water is available (Lin *et al.*, 2017), although both contribute to thermoregulation strategy.

Two simple temperature metrics encapsulate *T*_leaf_–*T*_air_ coupling relationships. The thermal offset (Δ*T*) describes the magnitude of difference between *T*_leaf_ and *T*_air_, and the thermal coupling strength (*β*) describes the slope of the relationship between *T*_leaf_ and *T*_air_ (Blonder and Michaletz, 2018; Blonder *et al.*, 2020). In nature, leaf thermal offsets can exceed ±15 °C (Salisbury and Spomer, 1964; Leuzinger and Körner, 2007; Blonder and Michaletz, 2018; Fauset *et al.*, 2018). Thermal coupling strength classifies plant thermoregulatory state into three categories: poikilothermy (*β* ≈ 1), limited homeothermy (*β* < 1) and megathermy (*β* > 1) (Blonder *et al.*, 2020; Cavaleri, 2020). Blonder *et al.* (2020) demonstrated that both Δ*T* and *β* can differ with environment across a range of *T*_air_ values in plant species from contrasting North American biomes. Specifically, at cool *T*_air_, species from temperate forests and meadows exhibit limited homeothermy [they have *T*_leaf_ warmer than *T*_air_ (negative Δ*T*)], but at warm *T*_air_, *T*_leaf_ is cooler than *T*_air_ and *β* < 1. In contrast, those from subalpine meadows were often poikilothermic, but sometimes exhibited megathermy with positive Δ*T* when *T*_air_ was high. High desert species were more variable but frequently exhibited megathermy with generally large positive Δ*T*, especially when *T*_air_ was high.

Plants from hot, arid environments, such as deserts, are frequently exposed to very high *T*_air_ and may not have water available to transpire freely to reduce *T*_leaf_ (Cook *et al.*, 2021), such that many desert plants tolerate rather than avoid high *T*_leaf_ (Curtis *et al.*, 2016). A common adaptation in desert plants is small leaf area to minimize overheating, reduce transpiration and increase water-use efficiency, but some large-leafed desert plants can maintain much higher transpiration rates and relatively low *T*_leaf_ (Smith, 1978). Many leaf traits contribute to mediating large thermal offsets (Guo *et al.*, 2022). For example, in tropical plants, *T*_leaf_ readily exceeds *T*_air_ (Manzi *et al.*, 2024); however, structural leaf traits are not necessarily individually related to Δ*T*. For example, in tropical shrubs and herbs, no relationship was found between Δ*T* and leaf area, leaf mass per area or leaf thickness (Pedraza, 2024). Data from dry temperate and tropical trees support the idea that transpirational cooling can be a strategy used to improve net carbon gain by avoiding leaf mortality or by maintaining temperature homeostasis near the optimal temperatures for photosynthesis (Slot and Winter, 2017; Drake *et al.*, 2018). Alpine plants tend to have strategies that aim to retain heat, because their environment is typically limited by cold temperatures, and *T*_leaf_ can exceed *T*_air_ by 15 °C or more, especially in short-statured plants (Salisbury and Spomer, 1964). Thus, high temperatures that occur during heatwaves and extremely hot days will result in unequal thermal exposure among different plant species, especially those with different thermoregulation strategies.

Determining the drivers of variation in thermal coupling in high-temperature conditions should therefore be a priority for understanding impacts to plant performance in the context of global change. Although theoretical predictions of how leaf thermoregulation should vary with environments have been established for decades, empirical studies addressing this question are rare. A recent field study along a temperature and precipitation gradient showed that plants from hotter sites showed greater transpirational cooling and that physical leaf traits were important for maintaining thermoregulation (Zhou *et al.*, 2023). To our knowledge, there have not been empirical studies in controlled environments that explore how common-grown species adapted to very different biomes vary in their leaf thermodynamic properties, and the structural or physiological drivers of leaf thermoregulation.

Our overarching goal was to determine how leaf characteristics facilitate or constrain leaf thermoregulation via thermal coupling. Here, we determined Δ*T* and *β* in 15 plant species, 5 from each of three contrasting biomes (alpine, desert and coastal temperate) in benign and high air temperatures in a controlled-environment glasshouse experiment. We then tested whether leaf structural traits and stomatal conductance were associated with leaf thermoregulation. We hypothesized that species originating from biomes with more extreme climates (alpine and desert) would have greater thermoregulatory capacity than those from more benign climates (coastal temperate). This difference would reflect varying combinations of leaf traits with stomatal strategy. We expected that plants with relatively small and less succulent leaves (i.e. low water content, thinner) might be closer to *T*_air_ and that plants with conservative (i.e. lower and/or less dynamic) *g*_sw_ would be most limited in their ability to thermoregulate. Assessment of the proximal causes of variation in plant thermoregulation in diverse species in controlled conditions will contribute to an improvement in our understanding of plant thermal sensitivity and vulnerability during heat extremes in nature.

## MATERIALS AND METHODS

### Information on species, growth conditions and origin biome

Five native Australian plant species that each originated from one of three contrasting biomes were chosen to be grown in common conditions in glasshouses at The Australian National University, Canberra, ACT, Australia. The 15 species cover seven families and four growth forms (Table 1). A simple phylogenetic tree of the study species is shown in Supplementary Data Fig. S1.

**Table 1.** List of the 15 species studied, including their biome of origin, taxonomic family, general growth form and origin of plant material used in the experiment.

Species	Biome	Family	Growth form	Plant material
Eucalyptus pauciflora	Alpine	Myrtaceae	Tree	Nursery
Leptorhynchos squamatus	Alpine	Asteraceae	Forb	Nursery
Oxylobium ellipticum	Alpine	Fabaceae	Shrub	Seedbank
Ranunculus graniticola	Alpine	Ranunculaceae	Forb	Nursery
Xerochrysum subundulatum	Alpine	Asteraceae	Forb	Nursery
Acacia binervata	Temperate	Fabaceae	Tree–shrub	Seedbank
Acacia longifolia	Temperate	Fabaceae	Tree–shrub	Seedbank
Backhousia myrtifolia	Temperate	Myrtaceae	Tree	Seedbank
Melaleuca hypericifolia	Temperate	Myrtaceae	Tree–shrub	Nursery
Pittosporum undulatum	Temperate	Pittosporaceae	Tree–shrub	Nursery
Acacia aneura	Desert	Fabaceae	Tree–shrub	Seedbank
Acacia salicina	Desert	Fabaceae	Tree–shrub	Seedbank
Dodonaea viscosa	Desert	Sapindaceae	Shrub	Seedbank
Eucalyptus largiflorens	Desert	Myrtaceae	Tree	Seedbank
Flindersia maculosa	Desert	Rutaceae	Tree	Seedbank

Plants used in the experiment were germinated between August and December 2020 from seed accessions obtained from the Australian National Botanic Gardens Seed Bank and the Australian Botanic Gardens Australian PlantBank. Seed accessions were collected originally within a 50 km radius within three distinct biomes (temperate: Wollongong, NSW; alpine: Kosciuszko National Park, NSW; and desert: Bourke, NSW) and were stored in these facilities for <20 years. Mean climatic parameters of these origin biomes are provided in Table 2. Some species had poor seed germination rates and were purchased as seedings from Monaro Native Tree Nursery, NSW and Bodalla Nursery, NSW at ~3 months old, which were then acclimated and grown in the same conditions as plants grown from seed (Table 1). Additional information is provided by Harris *et al.* (2024). The plants were grown in common garden well-watered conditions (watered to field capacity daily) in shade houses. Plants were transplanted in August 2021 to large pots (150–200 mm in diameter and ≥200 mm in depth) based on their individual size. The plants had grown for ~12–18 months before being moved to glasshouse conditions for this experiment in January–February 2022 (Austral summer) and ranged in size from 0.15 to 1.5 m in height at the time of the experiment. We used five replicate plants of each species for the temperature experiment. The plants were watered to saturation in the morning, before applying the temperature treatments to plants in controlled glasshouse rooms from 12.00 to 15.00 h, where the initial 30-min period from 12.00 to 12.30 h was considered temperature equilibration time. Plants did not show visual signs of water stress (i.e. they did not run out of water during the treatment phase) and were re-watered after the treatments.

**Table 2.** Environmental conditions of biomes of origin based on averages of downsampled long-term (1981–2010) climate data from CHELSA v.2.1 database (Karger *et al.*, 2017) using field locations for these alpine, temperate and desert biomes (Briceño *et al.*, 2024).

Biome	MAT (°C)	MinT (°C)	MaxT (°C)	*T*_range_ (°C)	MAP (mm)
Alpine	4.5	−5.2	16.5	22.7	1764
Temperate	16.5	7.4	24.6	17.2	1285
Desert	20.2	4.5	36.0	31.5	332

Abbreviations: MAP, mean annual precipitation; MAT, mean annual temperature; MaxT, mean maximum temperature of the warmest month; MinT, mean minimum temperature of the coldest month; *T*_*r*ange_, MaxT − MinT).

### Temperature treatments

**Fig. 1.**
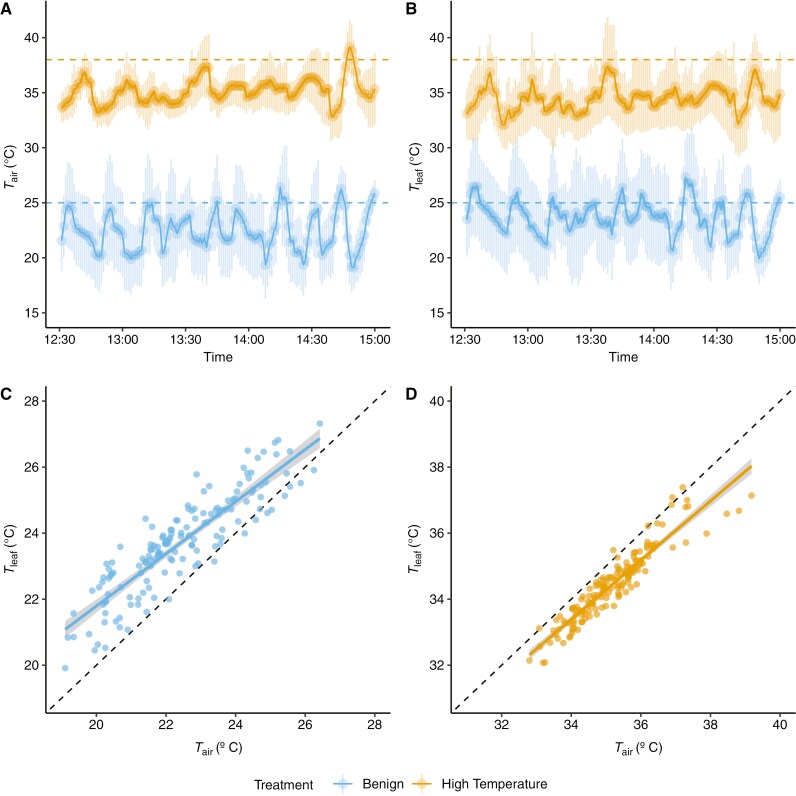
Temperature profiles and relationships between air temperature (*T*_air_) and leaf temperature (*T*_leaf_) in the glasshouse experiment. (A, B) Canopy *T*_air_ (A) and *T*_leaf_ (B) profiles over time across all plants for the benign- and high-temperature treatments during the experiment. Coloured dashed lines represent the glasshouse set temperatures for each treatment. Data shown are means ± 95 % confidence intervals across 6 days of measurement. (C, D) Relationship between *T*_air_ and *T*_leaf_ in the benign treatment (C) and the high-temperature treatment (D). Black dashed lines represent an isometric relationship, and coloured solid lines are simple linear regressions ± 95 % confidence intervals.

Two temperature treatments referred to as ‘benign’ and ‘high temperature’ were applied sequentially using controlled-temperature glasshouse rooms. The benign glasshouse room was set to 25 °C (06.00–20.00 h) during the day, and the high-temperature glasshouse room was set to 38 °C. The high temperature of 38 °C was chosen as a temperature that would be sufficiently stressful, but not lethal, for all species (Harris *et al.*, 2024). Both treatment glasshouses were set to and 16 °C overnight (20.00–06.00 h). All plants (*n* = 75) were moved from their shade house to the benign room 14 days before the experiment began, to allow for acclimation to the higher-light environment. Preliminary tests of high-temperature treatment duration effects on plant temperatures showed that Δ*T* (calculated as *T*_leaf_ − *T*_air_) of ten test plants averaged over 2.5 h was not different from longer periods of 4 or 6 h of high-temperature exposure, hence the 2.5 h duration (i.e. 12.30–15.00 h) was used. The experiment was conducted over six separate days (three for each treatment), where 30 plants were measured at a time. *T*_air_ at canopy level averaged across each of the plants during the treatments over 2.5 h was ~23.2 °C in the benign treatment and 35.7 °C in the high-temperature treatment (Fig. 1). Glasshouse conditions during the treatments were as follows for benign: temperature (*T*_gh_) = 26.3 ± 0.6 °C, relative humidity (RH) = 30.5 ± 4.5 % and vapour pressure deficit (VPD_air_) = 2.4 ± 0.5 kPa; and for high temperature: *T*_gh_ = 38.5 ± 0.4 °C, RH = 23.8 ± 3.4 % and VPD_air_ = 6.3 ± 0.3 kPa (full details are in Supplementary Data Table S1).

### Leaf temperature measurements

Leaf temperature (*T*_leaf_) measurements were taken using data loggers (Onset HOBO UX120-014M; Onset Computer Co., Bourne, MA, USA) and type-T thermocouples on mature, fully expanded, sun leaves emerging from the main stem or central part of each plant. Each thermocouple for measuring *T*_leaf_ was attached firmly to the underside of a leaf using porous surgical tape, and the thermocouple wire was supported by malleable wire on the stem to hold it in position without altering the natural leaf position. A second thermocouple was also anchored to the main stem of each plant, with the thermocouple tip open to air shielded from direct sunlight. This pairing enabled us to measure *T*_air_ immediately adjacent to the thermocouple measuring *T*_leaf_. The data loggers recorded temperature at 1-min intervals from 12.00 to 15.00 h.

Plants that were wired for temperature measurements in benign conditions were then transferred to the high-temperature conditions 2–4 days later, at ~11.00 h. Wherever possible, after the benign treatment the thermocouples were left in position, meaning that the *T*_leaf_ and *T*_air_ measurements were taken from the same location in both treatments. If a leaf began to discolour or if the thermocouple detached and could not be reattached easily, the thermocouple was moved to the nearest healthy, mature leaf to capture a similar microclimate. Logged measurements were trimmed to above 16 °C for the benign treatment and 31 °C for the high-temperature treatment, to exclude data when glasshouse evaporative coolers were active, because air circulation patterns during the active heating/cooling cycles introduced high variance and did not address our scientific questions (~10 % of the data; Fig. 1A, B). We calculated the thermal offset (Δ*T*), as *T*_leaf_ − *T*_air_ (in degrees Celsius) between 12.30 and 15.00 h to allow for temperatures to equilibrate. Negative values of Δ*T* occur when leaves are cooler than air and positive values of Δ*T* occur when leaves are warmer than air (Fig. 1C, D). We also calculated thermal coupling strength (*β*) as the slope of the relationship between *T*_leaf_ and *T*_air_ at 30-min intervals, following Blonder *et al.* (2020). Mean temperature responses per species are shown in Supplementary Data Table S2.

### Stomatal conductance

The stomatal conductance to water (*g*_sw_; in moles per metre squared per second) of light-adapted leaves was measured using a porometer–fluorometer (LI-600; LI-COR Biosciences, Lincoln, NE, USA). Transpiration (*E*) correlated strongly with *g*_sw_ (Pearson’s *r* = 0.90); therefore, we report only the *g*_sw_ results. The *g*_sw_ was measured on the same leaf that had the *T*_leaf_ thermocouple attached wherever possible, and species with small or compact leaves had *g*_sw_ measured on the closest mature, similar leaf. There were 18 (of 75) plants for which *g*_sw_ could not be measured owing to small leaf size; therefore, there were *n* = 57 plants in each temperature treatment for which there were a complete set of leaf traits for principal components analysis. Measurements of *g*_sw_ were taken twice between 13.30 and 14.30 h, after the plants had been exposed to the treatments for ≥1.5 h, and the average of both measurements was used.

### Leaf structural traits

After completing *T*_leaf_ and *T*_air_ measurements for both benign and high-temperature treatments, the same leaves that were measured for temperature were excised carefully from the plant to measure structural traits. Leaf wet mass (in milligrams) was measured with a precision balance (ML203T; Mettler-Toledo, Columbus, OH, USA), then leaf area (LA; in centimetres squared) using the *leafscan* app (Anderson and Rosas-Anderson, 2017), and leaf width (LW; in millimetres) and leaf thickness (LT; in millimetres) with precision callipers. The leaves were then placed in an oven at 60 °C for ≥72 h to dry completely. Dried leaves were then weighed for dry mass (in milligrams), allowing the calculation of leaf water content [LWC; (wet mass − dry mass)/wet mass], leaf density [LD; dry mass/(LA × LT); in grams per centimetre cubed], leaf mass per area (LMA; dry mass/leaf area; in kilograms per metre squared), and leaf dry matter content (LDMC; dry mass/wet mass; in kilograms per kilogram). Summary statistics for individual traits are shown in Supplementary Data Table S3.

### Thermal time constant

We calculated the theoretical leaf thermal time constant (*τ*; in seconds) as a mechanistic composite trait that links leaf traits to time-dependent decoupling of *T*_leaf_ from ambient conditions in the absence of thermoregulation via latent heat flux (Michaletz *et al.*, 2015, 2016; Bison and Michaletz, 2024).


$$\begin{array}{l} \tau =\;\varphi \times LMA\times \left(\frac{c_{p,w}}{LDMC\times h}+\frac{c_{p,d}-c_{p,w}}{h}\right) \end{array}$$

Values for parameters (*φ*, *c*_*p*,*w*_ and *c*_*p*,*d*_) were as defined by Bison and Michaletz (2024), i.e. *φ* (the ratio of projected to total leaf area) was taken to be 0.5, specific heat capacities *c*_*p*,*w*_ and *c*_*p*,*d*_ were taken as 4181 and 2814 J kg^−1^ K^−1^, respectively, and *h* is a heat transfer coefficient (in watts per metre squared per kelvin) that depends on leaf width (Michaletz *et al.*, 2016). Small values of *τ* represent leaves that change temperature rapidly in response to environmental temperature changes, and large values correspond to leaves that respond slowly. For additional information, see Supplementary Data Appendix S1.

### Statistical analyses

To test the nature of thermal decoupling (Δ*T* and *β*) in benign and high-temperature treatments across different species originating from the three biomes, we fitted linear mixed-effects regression (LMER) models. The temperate biome species and benign-temperature treatment were used as reference levels, and all models contained random effect (intercept) terms for growth form, species nested within taxonomic family, and plant identity to account for repeated measures on the same plants. The LMER models were fitted with either Δ*T* or *β* as the response variable, with treatment, biome and their interaction as categorical fixed effects.

To determine the effects of the combined leaf traits and their interaction with biome on Δ*T* and *β*, LMER models were fitted initially to the benign and high-temperature treatments separately. We generated composite leaf traits in two ways: principal components analysis and the thermal time constant (*τ*). For principal components analysis, we included the five passive leaf traits (LA, LW, LT, LWC and LD) and the active leaf trait (*g*_sw_), which generated two major axes of variation (PC1 and PC2; Supplementary Data Table S4). For these models, the random effects of species and growth form explained near-zero variance owing to redundancy with the leaf traits; therefore, simplified linear models were fitted to the benign and high-temperature treatments separately to determine the effects of composite leaf traits on Δ*T* and *β*. These models included two-way interactions between either PC1 and PC2 or *τ* with biome. We applied type III ANOVAs with Satterthwaite’s degrees of freedom (d.f.) to LMER models, followed by Tukey’s honest significant differences *post hoc* tests with Kenward-Roger’s d.f. for reporting. *Post hoc* tests compared pairwise differences among combinations of biome and treatment. The 95 % confidence intervals were obtained using non-parametric bootstrapping with the *mean_cl_boot* function from *Hmisc* (Harrell, 2019). All data analyses were conducted in R v.4.3.1 (R Core Team, 2023) using *lme4* (Bates *et al.*, 2015), *performance* (Lüdecke *et al.*, 2021), *emmeans* (Lenth, 2023), *factoextra* (Kassambara and Mundt, 2020) and *tidyverse* R packages (Wickham *et al.*, 2019).

## RESULTS

### Leaf thermal decoupling depends on both biomes and temperature treatments

**Fig. 2.**
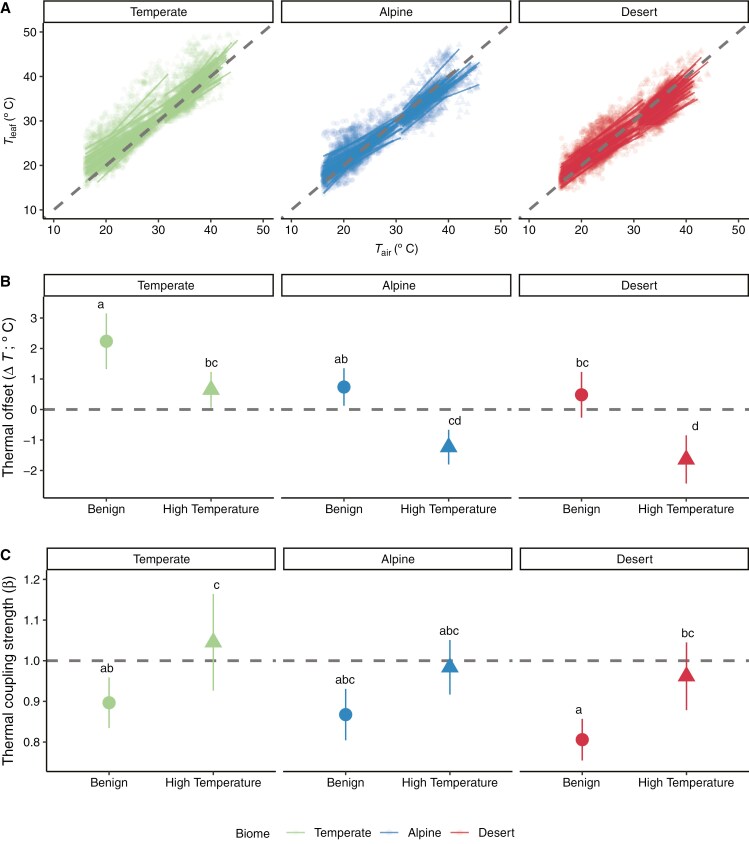
Canopy leaf and air temperature relationships, thermal coupling strength and thermal offsets among species from three biomes and two temperature treatments. (A) The overall raw data for relationships between *T*_leaf_ and *T*_air_, where linear regressions are fitted to individual plants. (B) Mean thermal offset (Δ*T*), which is the magnitude of the difference, *T*_leaf_ − *T*_air_. (C) Mean thermal coupling strengths (*β*), which is the slope of the relationship between *T*_leaf_ and *T*_air_, calculated at 30-min intervals. *β* > 1 indicates megathermy, *β* < 1 indicates limited homeothermy, and *β* ≈ 1 indicates poikilothermy. Data shown are means ± s.e.m. The grey lines for all panels (isometric, *β* = 1, Δ*T* = 0) indicate when *T*_air_ and *T*_leaf_ are equivalent.

We hypothesized that species originating from the more extreme alpine and desert climates would have greater thermoregulation tendency (Δ*T* differing from zero and *β* differing from one, exhibiting either megathermy or limited homeothermy) than those originating from the more benign temperate climate. The overall effect of treatment on Δ*T* was significant (Table 3), whereby high-temperature conditions resulted in significantly more negative Δ*T* (cooler leaves) than the benign treatment (Fig. 1). There was substantial variation in *T*_leaf_ along the *T*_air_ continuum both within and among biomes (Fig. 2A). On average, Δ*T* was positive for temperate species in both benign (1.99 ± 1.30 °C) and high-temperature (0.60 ± 0.91 °C) treatments (Fig. 2B). For both alpine and desert species, Δ*T* was positive in benign (alpine, 0.63 ± 1.01 °C; desert, 0.50 ± 1.05 °C) and negative in high-temperature (alpine, −1.25 ± 0.77 °C; desert, −1.66 ± 0.92 °C) treatments.

**Table 3.** Type III ANOVA outputs from linear mixed-effects regression (LMER) models that test the contributions of temperature treatment and biome on thermal offset (Δ*T*) and thermal coupling strength (*β*). *P < 0.05. Random effects are reported from the LMER summary.

Fixed effect	*F*	d.f.	*P*-value	Random effect	s.d.	Variance (%)
Response: thermal offset (Δ*T*)						
Treatment	77.893	1,72	<0.001*	Species	<0.001	0.0
Biome	22.332	2,45	<0.001*	Family	0.430	6.6
Treatment × biome	0.543	2,72	0.584	Growth form	0.665	15.8
				Plant identity	0.667	15.9
*R* ^2^ = 0.545				Residual	1.313	61.7

Response: thermal coupling strength (*β*)						
Treatment	27.244	1132	<0.001*	Species	0.033	3.5
Biome	2.230	2,7	0.181	Family	0.052	8.7
Treatment × biome	0.205	2132	0.815	Growth form	0.009	0.3
				Plant identity	<0.001	0.0
*R* ^2^ = 0.190				Residual	0.165	87.6

The effect of biome on Δ*T* was significant (Table 3), with alpine and desert species having ~1.2 °C cooler leaves than temperate species in both treatments (Fig. 2B). However, there were no significant interactions between biome and treatment (Table 3), such that the magnitude of difference in Δ*T* across biomes was consistent in both treatments (Fig. 2B). *Post hoc* tests revealed that pairwise temperature treatment differences in Δ*T* were significant within each biome (Fig. 2B; Supplementary Data Table S5). The temperate species were significantly different from desert species in either benign or high temperature and different from alpine species in high temperature (Fig. 2B; Supplementary Data Table S5).

Thermal coupling strength (*β*) was significantly higher in the high-temperature treatment, but not significantly different among biomes (Table 3; Fig. 2C). Species from all biomes typically exhibited limited homeothermy (*β* < 1) in benign conditions, but at high temperature, on average, temperate species exhibited megathermy (*β* > 1), whereas alpine and desert species exhibited poikilothermy (*β* ≈ 1) (Fig. 2C). *β* differed significantly between treatments in only the temperate and desert species, and the only other significant contrast was the temperate species in high temperatures compared with desert species in benign conditions (Fig. 2C; Supplementary Data Table S5).

**Fig. 3.**
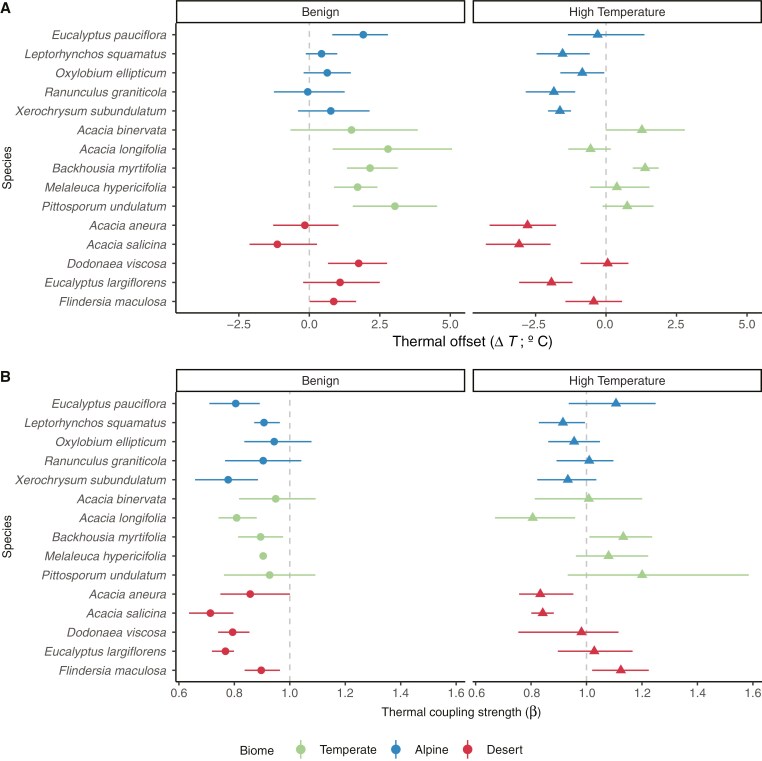
Thermal coupling parameters across species. Thermal offsets (Δ*T*; A) and thermal coupling strength (*β*; B) in benign (left) and high-temperature (right) treatments for each of 15 species originating from three biomes. Data shown are means ± bootstrapped 95 % confidence intervals. The dashed grey line at Δ*T* = 0 and *β* = 1 indicates when *T*_air_ and *T*_leaf_ are equivalent.

Taxonomic and growth form differences explained relatively small proportions of variance (in both temperature treatments, for both Δ*T* and *β*) beyond that explained by biome (Table 3). Across species, Δ*T* showed similar patterns in both temperature treatments (Fig. 3), with a few notable exceptions. *Acacia longifolia* (temperate) had the highest Δ*T* among temperate species in benign conditions but the lowest Δ*T* in high-temperature conditions (Fig. 3). *Eucalyptus largiflorens* (desert) also shifted from positive Δ*T* in benign conditions to a strongly negative Δ*T* in high-temperature conditions (Fig. 3). The most negative values of Δ*T* were achieved by two desert *Acacia* species, *Acacia salicina* and *Acacia aneura*. Both these species could cool their leaves below *T*_air_ by >3 °C in high-temperature conditions; >1 °C greater cooling than any other species tested.

### Leaf traits can moderate thermoregulation

Species-level leaf traits are shown in Supplementary Data Fig. S2, and the relationships of individual leaf traits and thermal coupling are shown in Supplementary Data Fig. S3. Given the strong effect of temperature treatment on Δ*T* and *β*, we analysed the effects of composite leaf traits on thermal coupling in each treatment separately. Biome was accounted for in all models and was significant in all cases except *β* at high temperature, indicating that differences in biome contributed to thermoregulatory differences indirectly (Table 4).

**Table 4.** ANOVA outputs from linear regression models that test the contributions of biome and leaf traits as principal components (PC1 and PC2) or as a composite thermal time constant (*τ*) on thermal offset (Δ*T*) and thermal coupling strength (*β*) separately in benign- and high-temperature conditions. *P < 0.05.

	Thermal offset (Δ*T*)				Thermal coupling strength (*β*)			
	Benign		High temperature		Benign		High temperature	
Fixed effects	*F*	*P*-value	*F*	*P*-value	*F*	*P*-value	*F*	*P*-value
Biome	6.178	0.004*	23.349	<0.001*	4.076	0.023*	1.512	0.231
PC1	5.730	0.021*	25.554	<0.001*	1.938	0.170	4.207	0.046*
PC2	0.489	0.488	0.847	0.362	0.787	0.380	0.061	0.807
Biome × PC1	2.934	0.063	0.115	0.892	1.194	0.312	0.454	0.638
Biome × PC2	0.442	0.645	1.041	0.361	1.732	0.188	0.019	0.981
Biome	6.000	0.005*	19.845	<0.001*	3.732	0.031*	1.575	0.217
*τ*	0.225	0.638	0.015	0.902	0.067	0.797	0.006	0.940
Biome× *τ*	3.996	0.024*	7.091	0.002*	0.369	0.693	2.215	0.120

**Fig. 4.**
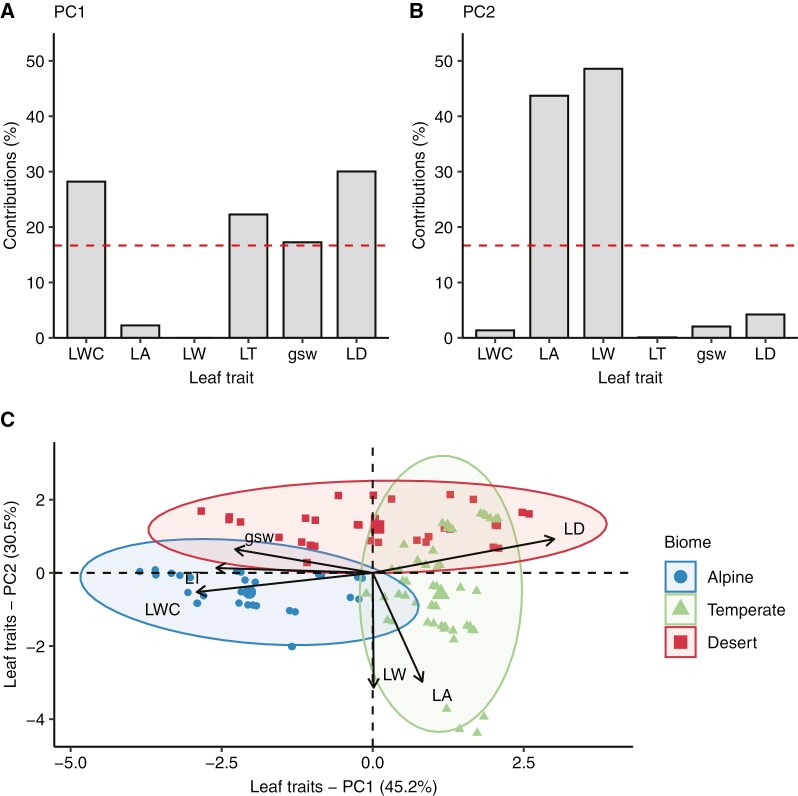
Principal components analysis of six leaf traits: leaf water content (LWC), leaf area (LA), leaf thickness (LT), leaf width (LW), stomatal conductance to water (*g*_sw_) and leaf density (LD). (A, B) Contributions of each leaf trait to PC1 (A) and PC2 (B) major axes. Red dashed line corresponds to the expected value if contributions were uniform among traits, where grey bars that are higher than the red line indicate dominant variables to that principal component. (C) Principal component space of dominant PC1 and PC2 axes that together explain 75.7 % of the variance in the leaf traits. Coloured ellipses represent the 95 % confidence space for each biome.

The composite leaf trait major axis (PC1) can be interpreted as an axis from negative values representing thick, less dense leaves with high water content and high stomatal conductance to positive values increasing towards thin, dense leaves with low water content and low stomatal conductance (Fig. 4A, C). The other dominant composite leaf trait axis (PC2) can be interpreted as a continuum from negative values representing wider, larger leaves to positive values increasing towards narrower, smaller leaves (Fig. 4B, C). The three biomes formed generally distinct clusters in principal component space. The leaves of alpine species were defined by negative PC1 (thick, less dense, high water content and high *g*_sw_) and a narrow range of slightly negative PC2 (Fig. 4C). The leaves of temperate species were defined by positive PC1 (thin, dense, low water content and low *g*_sw_) but spanned a wide range along PC2 (from broad and large to narrow and small) (Fig. 4C). The leaves of desert species covered a wide range of PC1, but all were positive along PC2 (small and narrow leaves) (Fig. 4C).

**Fig. 5.**
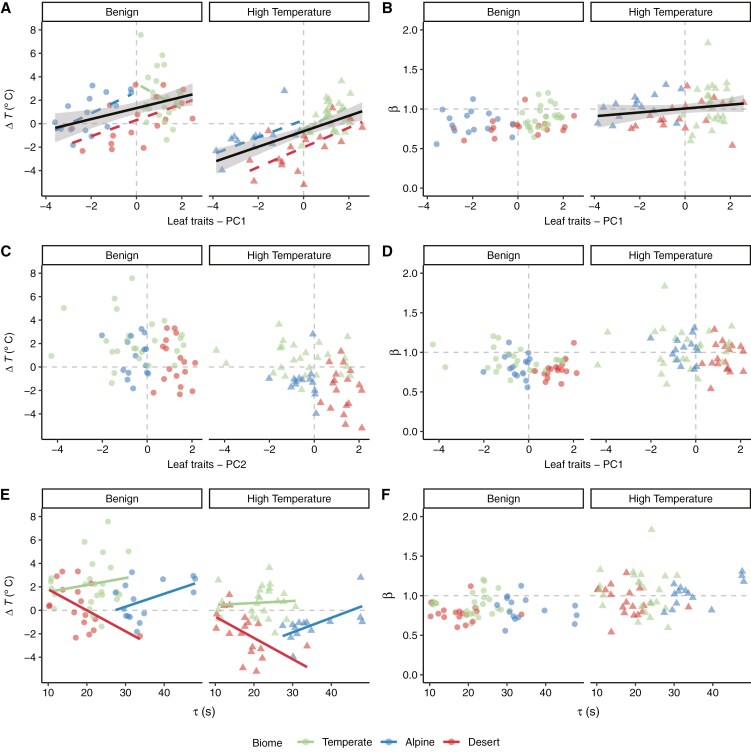
Relationships between leaf thermal coupling and composite leaf traits from principal components analysis (PC1 and PC2 axes) and thermal time constant (*τ*). (A, B) Thermal offset (Δ*T*; A) in relationship to leaf traits PC1 and thermal coupling strength (*β*; B) in relationship to leaf traits PC1 under benign and high-temperature treatments. (C, D) Δ*T* (C) and *β* (D) in relationship to leaf traits PC2. (E, F) Δ*T* (E) and *β* (F) in relationship to τ. Raw data are shown, and linear regressions are overlayed where relationships between trait and thermal coupling are significant (solid where *P* < 0.05 and dashed where *P* < 0.1) overall (black) or interact with biome (coloured). Corresponding model outputs are shown in Table 4. The dashed grey horizonal lines at Δ*T* = 0 and *β* = 1 indicate when *T*_air_ and *T*_leaf_ are equivalent, and the dashed grey vertical lines in A–D show PC1 = 0 and PC2 = 0.

Thermal offset (Δ*T*) increased significantly as PC1 increased in both treatments (Table 4), i.e. leaves that were thin, dense and had low water content and relatively low *g*_sw_ characteristics tended to be warmer than air (Fig. 5A). Thermal coupling strength (*β*) increased significantly as PC1 increased in the high-temperature treatment (Table 4), i.e. leaves that were thicker, less dense and had high water content and high *g*_sw_ exhibited limited homeothermy, whereas leaves that were thinner, denser and had low water content and low *g*_sw_ exhibited megathermy (Fig. 5B). There was a marginally non-significant interaction between PC1 and biome (Fig. 5A, C; Table 4). Neither Δ*T* nor *β* was significantly related to PC2 (Fig. 5C, D; Table 4). The relationship between Δ*T* and thermal time constant (*τ*) was not significant overall, but did differ significantly among biomes (Fig. 5E; Table 4). Desert species had a negative relationship between Δ*T* and *τ*, whereas temperate and alpine species had a positive relationship, and these patterns were consistent in both treatments (Fig. 5E; Table 4). Thermal coupling strength (*β*) showed no significant relationships with *τ* or the interaction between *τ* and biome in either treatment (Fig. 5F; Table 4).

## DISCUSSION

Here, we have shown that these diverse plant species that originate from contrasting biomes differ clearly in their thermoregulation in both benign and high-temperature conditions. Our hypotheses about the drivers of variation in plant thermoregulation were largely supported: variation in *T*_leaf_, Δ*T* and *β* during high temperatures was dependent on origin biome and composite leaf traits, especially leaf water content and *g*_sw_. Different species exposed to nearly identical conditions will reach different *T*_leaf_ owing to the unique interactions of their leaf properties with the environment (Perez and Feeley, 2020). Thus, understanding the sources of variation in *T*_leaf_ is essential: empirical data inform leaf energy budget theory and more accurate predictive models of *T*_leaf_ (Michaletz *et al.*, 2015; Blonder *et al.*, 2020; Kearney and Leigh, 2024).

### Plants from extreme climates can thermoregulate more effectively

Our hypothesis that species originating from biomes with more extreme climates would have greater thermoregulatory tendency than those from more benign climates was generally supported. That is, the adaptations a plant has to the environmental conditions of its biome of origin explain thermoregulation even in common conditions. Temperate species had leaves that were almost always warmer than air, whereas the leaves of alpine and desert species were equivalent to *T*_air_ in benign conditions but often much cooler than air at high temperatures. Species from all biomes showed limited homeothermy (*β* < 1) in benign conditions, but at high temperature, most exhibited poikilothermy (*β* ≈ 1).

Leaf thermoregulation is thought to originate from selection on leaf traits to maximize carbon gain in their environment (Michaletz *et al.*, 2016). That is, plants will maintain *T*_leaf_ within an optimal range for photosynthesis via variable stomatal opening to thermoregulate actively at an approximate crossover point when *T*_leaf_ reaches ~27–30 °C and Δ*T* becomes negative (Michaletz *et al.*, 2016; Dong *et al.*, 2017). Experimental tests of the limited homeothermy hypothesis found that cotton (*Gossypium hirsutum*) exhibited poikilothermy until *T*_air_ reached 27 °C, then switched to limited homeothermy when *T*_air_ was 27–40 °C to maintain *T*_leaf_ = 27 ± 2 °C, when water was available for transpiration (Upchurch and Mahan, 1988). However, recent large-scale analyses of canopy temperatures generally do not support a hypothesis of universal limited homeothermy; rather, there is evidence for a diverse range of viable thermoregulation strategies (*β* range = 0.7–1.3) (Still *et al.*, 2022; Guo *et al.*, 2023; Manzi *et al.*, 2024). Our present study provides empirical support for these recent analyses at a smaller scale, where plants (on average) exhibited limited homeothermy in benign conditions, but a wide range of *β* in high temperatures.

### Potential links between leaf thermoregulation and photosystem heat tolerance

A common measure of photosystem heat tolerance is *T*_crit_, the critical temperature for photosystem II functional impairment and subsequent damage, which is derived from ramping assays of the temperature-dependent change in chlorophyll *a* fluorescence (Arnold *et al.*, 2021). Many of the species in the present study are known to differ in *T*_crit_ from field surveys (Briceño *et al.*, 2024) and controlled-environment experiments (Harris *et al.*, 2024). Drawing links between *T*_crit_ from these studies and thermoregulation from our study returns some unexpected outcomes. Notably, *Dodonaea viscosa* was one of the least heat-tolerant desert species in the aforementioned studies (controlled 25 °C environment, 39.9 ± 1.0 °C; field, 45.4 ± 0.5 °C), and in the present study it had the highest *T*_leaf_ and Δ*T* ≈ 0 °C in high-temperature conditions. The relatively low heat-tolerance threshold of the alpine *Eucalyptus pauciflora* (controlled 25 °C environment, 42.7 ± 1.7 °C; field, 39.0 ± 0.8 °C) and the temperate *Acacia binervata* (controlled 25 °C environment, 42.0 ± 1.5 °C), which both had positive Δ*T* values at high temperatures in the present study, is consistent with this pattern. The reverse is true for the desert *Acacia* species, which are both extremely heat tolerant (*A. salicina*: controlled 25 °C environment, 46.9 ± 0.8 °C; field, 49.6 ± 0.8 °C; and *A. aneura*: controlled 25 °C environment, 48.6 ± 0.9 °C; field, 53.0 ± 4.0 °C), and here were found to have the lowest *T*_leaf_ and negative Δ*T* values at high temperatures.

We advocate for testing the association of thermal coupling metrics and heat tolerance as a focus of future investigations. Initially, it appears counter-intuitive that a species should have adaptations to avoid and tolerate high temperatures concurrently. In an extremely hot and dry environment, plants may typically avoid the worst of heat stress through their structural leaf properties and evaporative cooling via transpiration. Yet, sustained dry conditions may render evaporative cooling an unviable option for avoiding heat, and high heat tolerance would become necessary (Gong *et al.*, 2023). A species that does not cool *T*_leaf_ below *T*_air_ (or only cools moderately), might indicate limited capacity for cooling or a high heat-tolerance threshold before initiating cooling. As such, we hypothesize that the tendency to cool *T*_leaf_ below *T*_air_ could be associated with higher heat tolerance in some species, but that it will probably also depend on water-use strategy.

### Composite leaf traits contribute to thermoregulation at high temperature

The dominant axis of variation (PC1) was defined by three structural leaf traits [leaf thickness (LT), leaf density (LD) water content (LWC)] and by the active leaf trait, stomatal conductance (*g*_sw_). PC1 correlated strongly with Δ*T* in benign conditions and with Δ*T* and β in high-temperature conditions. PC2, which was largely defined by leaf area (LA) and leaf width (LW) had relatively little consistent relationship with Δ*T* and *β* in either environment. LWC plays a key role in leaf thermoregulation and leaf economics (Michaletz *et al.*, 2015; Wang *et al.*, 2022). The specific heat capacity is positively correlated with the water content of leaves (Zhang *et al.*, 2025), and there is also a strong positive association between LWC and maximum *g*_sw_ (Zhou *et al.*, 2023). Water availability to plants is generally linked to the capacity to regulate *T*_leaf_ (Lambers and Oliveira, 2019; Cook *et al.*, 2021; Manzi *et al.*, 2024), and the transport and storage of water directly in leaf tissues can reduce heat loading while facilitating greater cooling (Zhou *et al.*, 2023). In desert species, thicker leaves (that often also have higher LWC) have slower heating response times relative to thinner leaves, resulting in lower *T*_leaf_ during temperature extremes, even in the absence of transpiration, but the effect is reduced for large leaves (Leigh *et al.*, 2012). Our study shows that leaf cooling is more effective in plants that have higher LWC and LT and lower LD in both temperature treatments.

Combinations of leaf functional and energy budget traits and environments across 41 species and seven sites along an elevation gradient showed that regression approaches achieved relatively low predictive power for Δ*T* and especially for *β* (Blonder *et al.*, 2020). In their study, the site environment played a more substantial role than commonly measured functional traits and energy balance traits, and interactions between traits and environment were relevant. Blonder *et al.* (2020) concluded that the low predictability of thermal coupling and the variation encountered at a given site indicate that a range of strategies will result in viable performance. Our results are consistent with these findings: variation in Δ*T* was more readily explained than *β*. The common environment approach we used highlights that both external environment (temperature treatment) and origin biome strongly influence Δ*T*, whereas only environmental conditions influence *β*.

The thermal time constant (*τ*) differed among plants from the different biomes. The alpine plants in this study had relatively large values of *τ*, indicating that they respond more slowly to environmental changes than the desert or temperate plants, which could be to buffer against the rapid environmental temperature fluctuations that occur naturally in the alpine biome (Körner, 2003). We also found that the relationship between *τ* and Δ*T* differed among biomes, being positive for temperate and alpine plants, such that leaves that respond relatively more slowly to environmental changes were warmer than air or at least cooled less effectively. In contrast, the leaves of desert plants that respond relatively more slowly to environmental changes were, nonetheless, far more effective at cooling below *T*_air_, especially in comparison to temperate plants. That is, for the same value of *τ*, Δ*T* differed by ≤4 °C between desert and temperate plants, which suggests that *g*_sw_ was the main driver of these differences, because it is not involved in the calculation of *τ*. The dynamic fluctuations of *T*_air_ in glasshouse conditions suggests that *T*_leaf_ might not frequently reach a steady state within the range of *τ*, hence delays in both leaf warming and cooling might influence the relationships between traits and thermoregulation. All plants had access to adequate water throughout the heat event and could have transpired freely; however, desert plants transpired far more than temperate plants in both temperature treatments. If the desert plants with larger *τ* values opened their stomata earlier to achieve high *g*_sw_ and did so for longer than temperate plants with larger *τ* values, that could explain why the leaves of these desert plants were much cooler than air and why relationships with Δ*T* differed between these biomes.

### Inherently low stomatal conductance limits evaporative cooling

We predicted that species with inherently low *g*_sw_ would be most limited in their thermoregulation. Generally, high *g*_sw_ strongly reduced Δ*T*, which was consistent across biomes except for temperate species in benign conditions. Stomatal conductance and LWC both play pivotal roles in enabling leaf cooling at high temperature, thus reducing *T*_leaf_ on acutely hot days and during heatwaves will clearly depend on water availability and water-use strategies (Drake *et al.*, 2018; Aparecido *et al.*, 2020; Cook *et al.*, 2021; Marchin *et al.*, 2022; Manzi *et al.*, 2024). The temperate species originate from a biome that is typically not water limited, yet these species appear to be more limited in their tendency for thermoregulation via regulating stomata. One potential explanation for this is the intricate link between temperature and VPD. Increases in VPD are a major concern with climate change, because it can also limit evapotranspiration by exacerbating water stress and forcing stomatal closure (Grossiord *et al.*, 2020). Although the relative humidity in our glasshouse experiment was generally low enough to enable cooling to take place (Mahan and Upchurch, 1988), VPD increased in the high-temperature treatment. The lower *g*_sw_ in the species originating from the coastal temperate biome probably responded to the high-temperature (with relatively high VPD) treatment by closing stomata more than alpine and desert species that are adapted to typically drier air.

An alternative explanation is that these coastal temperate species have intrinsically lower *g*_sw_ or slower stomatal response to high temperatures in comparison to species that originated from more extreme climates. For example, some desert and alpine species can open their stomata rapidly to optimize the trade-offs between carbon fixation, water loss and leaf thermoregulation during narrower windows of suitable conditions in these challenging environments (Knapp and Smith, 1988, 1991; Fernández-Marín *et al.*, 2020). Glasshouse experiments with plants originating from hot dry and hot wet habitats suggest that transpiration is greater in species from hot dry habitats that have sporadic rain (Lin *et al.*, 2017), supporting the idea that extreme climate is a driver of thermoregulation strategy. Similar to our study, those authors found that cooling via stomatal behaviour was more effective than passive leaf traits when water was sufficient (Lin *et al.*, 2017). We infer that high *T*_air_ in our high-temperature treatment probably increased *T*_leaf_ to a point that exceeded the heat load that most of the temperate species could dissipate via transpiration.

### Leaf thermoregulation is a complex plant–environment interaction

Plant species are often interpreted as being on a water stress avoidance–tolerance (isohydric–anisohydric) spectrum; however, rather than being a simple plant hydraulic trait, isohydrocity is a complex plant–environment interaction (Hochberg *et al.*, 2018). Leaf thermoregulation seems analogous to this complexity. There are many causes for leaf thermoregulation depending on the immediate environment of the plant. For example, thermoregulation can optimize photosynthesis, but it also plays a role in hydraulic maintenance; then at extreme temperatures, thermoregulation facilitates avoidance of heat damage in the absence of photosynthesis (Slot and Winter, 2017; Drake *et al.*, 2018; Fauset *et al.*, 2018; Guo *et al.*, 2022). Therefore, the balance among the available thermoregulation mechanisms depends on these dynamic plant–environment interactions (Guo *et al.*, 2023). Contrasting patterns of leaf thermoregulatory traits and strategies among provenances across tropical trees demonstrates that warm-adapted provenances are not necessarily less vulnerable to heat stress based on their operating temperatures and heat tolerance (Middleby *et al.*, 2024). Elucidating the mechanisms that underlie differences in thermoregulatory strategies of plants across different origin biomes will be essential empirical research for applications to plant breeding and management of wild populations. Determining the physical leaf and stem properties and the underlying genetic markers and mechanisms for stomatal responsiveness that contribute to variation in plant thermoregulation and plasticity therein could be used to identify and select on target traits (Fritz *et al.*, 2018).

## Conclusion

Advanced tools for rapidly estimating leaf size (Schrader *et al.*, 2021; Leigh, 2022) and predicting leaf temperatures based on biophysical modelling with microclimates and energy budgets are now available (Kearney and Leigh, 2024). The accuracy of predicted leaf temperatures requires capturing and understanding the diversity of functional leaf traits and stomatal conductance behaviour, which can have a large impact on *T*_leaf_ predictions (Perez and Feeley, 2020; Kearney and Leigh, 2024). Our study provides empirical evidence that species from contrasting biomes that are exposed to common conditions (benign or high temperature) will respond to the conditions by regulating *T*_leaf_ to different extents. We also identify that composite leaf traits explain variation in leaf thermoregulation among species. Our findings suggest that, beyond simple expectations of leaf size, species from a coastal temperate biome appear to possess a suite of thermoregulatory traits more likely to increase exposure to heat stress, particularly if combined with dry conditions, than those adapted to more extreme conditions. The increasingly extreme environmental conditions that are occurring during the Anthropocene are exerting significant pressure on plants in many regions to avoid, tolerate and acclimatize to higher temperatures. Further work should evaluate interactive effects among temperature, VPD and water availability to discern the impacts of these major global change factors on leaf thermoregulation.

## SUPPLEMENTARY DATA

Supplementary data are available at *Annals of Botany* online and consist of the following.

Appendix S1: thermal time constant notes. Figure S1: phylogenetic tree of the 15 species in the experiment. Figure S2: leaf trait values across species. Figure S3. relationships between leaf thermal coupling and six key leaf traits. Table S1: glasshouse environmental parameters. Table S2: summary statistics for each thermal trait. Table S3: summary statistics for each leaf trait. Table S4: principal components analysis variable loadings for leaf traits. Table S5: pairwise contrasts for Δ*T* and *β* among biomes and treatments.

mcaf080_suppl_Supplementary_Material

## Data Availability

Data and R code are available from the Figshare digital repository: https://doi.org/10.6084/m9.figshare.28741331.
