# Supplementary material for: Plants originating from more extreme biomes have improved leaf thermoregulation
Source: Ann Bot. 2025 May 4;136(1):199–213. doi: 10.1093/aob/mcaf080 (PMC12401887; doi:10.1093/aob/mcaf080)
Supplement: mcaf080_suppl_Supplementary_Material [file mcaf080_suppl_supplementary_material.docx]

***Supplementary Information***

**Plants originating from more extreme biomes have improved leaf thermoregulation**

Pieter A. Arnold, Monique J. White, Alicia M. Cook, Andrea Leigh, Verónica F. Briceño, Adrienne B. Nicotra

**Table of Contents**

**Appendix S1:** Thermal time constant notes.

**Figure S1:** Phylogenetic tree of the 15 species in the experiment.

**Figure S2:** Leaf trait values across species.

**Figure S3:** Relationships between leaf thermal coupling and six key leaf traits.

**Table S1:** Glasshouse environmental parameters.

**Table S2:** Summary statistics for each thermal trait.

**Table S3:** Summary statistics for each leaf trait.

**Table S4:** Principal Component Analysis (PCA) variable loadings for leaf traits.

**Table S5:** Pairwise contrasts for Δ*T* and β among biome and treatments.

***Appendix S1: Thermal time constant notes***

The theoretical leaf thermal time constant (τ; s) was calculated as a mechanistic composite trait that links leaf traits to time-dependent decoupling of *T*_leaf_ from ambient conditions in absence of thermoregulation via latent heat flux (Michaletz et al., 2016, Michaletz et al., 2015, Bison and Michaletz, 2024).

$$\tau= \varphi\cdot\mathrm{LMA}\cdot\left[ \frac{c_{p,w}}{\mathrm{LDMC}\cdot h}+\frac{c_{p,d}-c_{p,w}}{h} \right]$$

Leaf mass per area (LMA; dry mass / leaf area) was converted to kg m^-2^, and leaf dry matter content (LDMC; dry mass / wet mass) was converted to kg kg^-1^ for calculation of τ. Values for the other parameters (*ϕ*, *c_p,w_*, *c_p,d_*, *h*) were as defined by Bison and Michaletz (2024), i.e. *ϕ* (the ratio of projected to total leaf area) was taken to be 0.5, specific heat capacities *c_p,w_* and *c_p,d_* were taken as 4181 and 2814 J kg^-1^ K^-1^, respectively, and *h* is an heat transfer coefficient (W m^-2^ K^-1^) that depends on leaf width (Michaletz et al., 2016).

The heat transfer coefficient (*h*) was calculated as

$$h=\rho_{a}\cdot c_{p,a}\cdot g_{h}$$

where *ρ_a_* is the mass density of air (1.224 kg m^-3^), *c_p,a_* is the specific heat capacity of air (1007 J kg^-1^ K^-1^) and *g_h_* is boundary later conductance to heat, calculated as

$$g_{h}=\frac{1.5\cdot6.62\cdot\left[ \frac{U}{\mathrm{LW}} \right]^{0.5}}{1000}$$

where *U* is an assumed windspeed of 0.05 m s^-1^ (based on still glasshouse conditions), and LW is leaf width (sometimes denoted as *w*_e_ or *L*), following Bison and Michaletz (2024).

** Figure S1.** Phylogenetic tree of plausible relatedness among the 15 study species. Coloured circles on tips represent the origin biome: temperate = green, alpine = blue, and desert = red.

**Figure S2.** Leaf trait values across species. For each of 15 species originating from three biomes, **A)** leaf area, LA (mm^2^), **B)** leaf width, LW (mm), **C)** leaf thickness, LT (mm), **D)** leaf water content, LWC, **E)** leaf density, LD (g cm^-3^), **F)** stomatal conductance to water vapour, *g*_sw_ (mol m^-2^ s^-1^) under benign temperature treatment (left facet) and high temperature treatment (right facet). Data shown are means ± bootstrapped 95% confidence intervals.

**
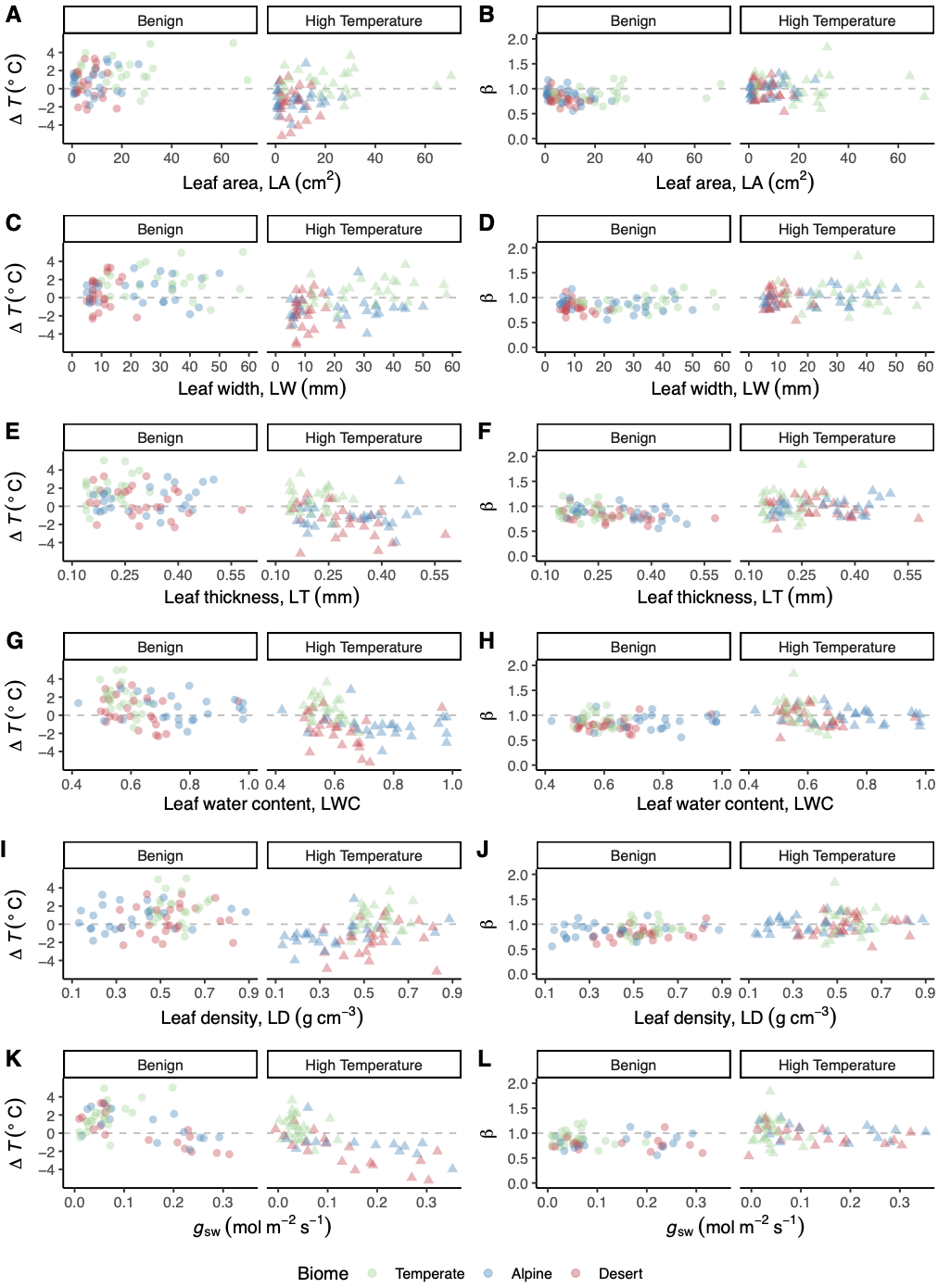
**

**Figure S3.** Relationships between leaf thermal coupling and six key leaf traits. The relationship between each of thermal offsets (Δ*T*; left column) and thermal coupling strength (β; right column) with **A-B)** leaf area, LA (cm^2^), **C-D)** leaf width, LW (mm), **E-F)** leaf thickness, LT (mm), **G-H)** leaf water content, LWC, **I-J)** leaf density, LD (g cm^-3^), and **K-L)** stomatal conductance to water vapour, *g*_sw_ (mol m^-2^ s^-1^) under benign temperature treatment and high temperature treatments (panel facets).

**Table S1.** Glasshouse environmental parameters: temperature, RH, and VPD values between 12:30-15:00 on each day of measurement of the experiment.

| **Date** | **Treatment** | ***T*_gh_ (°C)** | **RH (%)** | **VPD_air_ (kPa)** |
| --- | --- | --- | --- | --- |
| 24 Jan 2022 | Benign | 26.8 ± 0.7 | 28.6 ± 3.7 | 2.5 ± 0.5 |
| 31 Jan 2022 | Benign | 26.7 ± 0.7 | 25.3 ± 4.7 | 2.5 ± 0.5 |
| 04 Feb 2022 | Benign | 25.5 ± 0.4 | 37.6 ± 5.2 | 2.2 ± 0.5 |
| 28 Jan 2022 | High temperature | 38.5 ± 0.3 | 27.9 ± 3.9 | 6.2 ± 0.4 |
| 02 Feb 2022 | High temperature | 38.3 ± 0.4 | 28.5 ± 4.5 | 6.1 ± 0.4 |
| 07 Feb 2022 | High temperature | 38.7 ± 0.5 | 15.0 ± 1.7 | 6.5 ± 0.2 |

Note: Data reported is mean ± 95% confidence intervals from glasshouse thermostat data recorded every 5 minutes. Set temperatures were 25°C for benign and 38°C for high temperature treatments. *T*_gh_ = glasshouse air temperature, RH = relative humidity, VPD_air_ = vapour pressure deficit of the air.

**Table S2.** Summary statistics (mean ± SE) for each thermal trait measured in the experiment across species.

|  |  | **Benign** | | | | **High temperature** | | | |
| --- | --- | --- | --- | --- | --- | --- | --- | --- | --- |
| **Biome** | **Species** | ***T*_air_ (°C)** | ***T*_leaf_ (°C)** | **Δ*T* (°C)** | **β** | ***T*_air_ (°C)** | ***T*_leaf_ (°C)** | **Δ*T* (°C)** | **β** |
| Alpine | *Eucalyptus pauciflora* | 22.1 ± 1.61 | 24.0 ± 1.70 | 1.90 ± 1.02 | 0.81 ± 0.10 | 35.2 ± 0.94 | 34.9 ± 1.55 | –0.33 ± 0.92 | 1.10 ± 0.14 |
| Alpine | *Leptorhynchos squamatus* | 22.2 ± 1.59 | 22.6 ± 1.43 | 0.41 ± 0.66 | 0.91 ± 0.05 | 36.7 ± 1.44 | 35.1 ± 1.12 | –1.55 ± 0.76 | 0.91 ± 0.07 |
| Alpine | *Oxylobium ellipticum* | 21.3 ± 1.56 | 22.0 ± 1.42 | 0.78 ± 0.80 | 0.95 ± 0.11 | 34.1 ± 0.79 | 33.2 ± 0.96 | –0.86 ± 0.53 | 0.96 ± 0.12 |
| Alpine | *Ranunculus graniticola* | 21.6 ± 1.46 | 21.6 ± 1.43 | 0.01 ± 0.96 | 0.92 ± 0.11 | 34.5 ± 0.87 | 32.7 ± 0.97 | –1.83 ± 0.67 | 1.01 ± 0.12 |
| Alpine | *Xerochrysum subundulatum* | 21.4 ± 1.61 | 22.3 ± 1.49 | 0.93 ± 1.24 | 0.79 ± 0.10 | 34.7 ± 0.97 | 33.1 ± 1.02 | –1.63 ± 0.54 | 0.93 ± 0.13 |
| Temperate | *Acacia binervata* | 23.7 ± 1.51 | 25.2 ± 1.69 | 1.46 ± 1.46 | 0.95 ± 0.10 | 35.2 ± 1.01 | 36.4 ± 1.55 | 1.20 ± 1.05 | 1.02 ± 0.20 |
| Temperate | *Acacia longifolia* | 24.2 ± 1.82 | 26.9 ± 2.33 | 2.71 ± 1.58 | 0.80 ± 0.11 | 35.8 ± 0.94 | 35.3 ± 0.90 | –0.57 ± 0.66 | 0.83 ± 0.11 |
| Temperate | *Backhousia myrtifolia* | 22.2 ± 1.65 | 24.3 ± 1.46 | 2.05 ± 1.10 | 0.91 ± 0.09 | 36.1 ± 1.02 | 37.5 ± 1.45 | 1.37 ± 0.84 | 1.13 ± 0.15 |
| Temperate | *Melaleuca hypericifolia* | 23.1 ± 1.50 | 25.0 ± 1.75 | 1.89 ± 1.02 | 0.90 ± 0.08 | 35.8 ± 0.96 | 36.2 ± 1.40 | 0.37 ± 0.76 | 1.08 ± 0.15 |
| Temperate | *Pittosporum undulatum* | 21.4 ± 1.54 | 24.4 ± 1.75 | 3.07 ± 1.24 | 0.93 ± 0.11 | 35.4 ± 0.89 | 36.1 ± 1.39 | 0.76 ± 0.91 | 1.21 ± 0.27 |
| Desert | *Acacia aneura* | 21.3 ± 1.60 | 20.9 ± 1.40 | –0.35 ± 0.78 | 0.86 ± 0.10 | 34.2 ± 0.84 | 31.5 ± 0.80 | –2.76 ± 0.67 | 0.83 ± 0.11 |
| Desert | *Acacia salicina* | 24.8 ± 1.42 | 23.8 ± 1.46 | –0.98 ± 0.91 | 0.71 ± 0.07 | 35.1 ± 0.88 | 32.0 ± 1.03 | –3.09 ± 0.79 | 0.84 ± 0.13 |
| Desert | *Dodonaea viscosa* | 23.5 ± 1.60 | 25.3 ± 1.70 | 1.81 ± 0.95 | 0.79 ± 0.08 | 35.7 ± 0.86 | 35.8 ± 1.18 | 0.12 ± 0.75 | 0.98 ± 0.22 |
| Desert | *Eucalyptus largiflorens* | 22.6 ± 1.65 | 23.9 ± 1.82 | 1.27 ± 1.13 | 0.77 ± 0.07 | 35.4 ± 0.93 | 33.5 ± 1.14 | –1.92 ± 0.67 | 1.03 ± 0.15 |
| Desert | *Flindersia maculosa* | 21.6 ± 1.60 | 22.6 ± 1.42 | 0.97 ± 0.68 | 0.92 ± 0.07 | 34.5 ± 0.93 | 34.1 ± 0.99 | –0.43 ± 0.64 | 1.13 ± 0.13 |

*T*_air_ = air temperature, *T*_leaf_ = leaf temperature, Δ*T* = thermal offset, β = thermal coupling strength.

**Table S3.** Summary statistics (mean ± SE) for each leaf trait measured in the experiment across species.

|  |  |  |  |  |  |  | **Benign** | **High temperature** |
| --- | --- | --- | --- | --- | --- | --- | --- | --- |
| **Biome** | **Species** | **LA** | **LW** | **LT** | **LWC** | **LD** | ***g*_sw_** | ***g*_sw_** |
| Alpine | *Eucalyptus pauciflora* | 18.0 ± 2.24 | 35.5 ± 3.48 | 0.44 ± 0.02 | 0.64 ± 0.02 | 0.40 ± 0.04 | 0.04 ± 0.01 | 0.04 ± 0.01 |
| Alpine | *Leptorhynchos squamatus* | 0.80 ± 0.08 | 5.73 ± 0.32 | 0.20 ± 0.01 | 0.72 ± 0.02 | 0.39 ± 0.02 | NA | NA |
| Alpine | *Oxylobium ellipticum* | 0.93 ± 0.09 | 10.0 ± 1.83 | 0.18 ± 0.01 | 0.54 ± 0.04 | 0.69 ± 0.07 | NA | NA |
| Alpine | *Ranunculus graniticola* | 7.61 ± 0.55 | 38.2 ± 2.30 | 0.37 ± 0.03 | 0.76 ± 0.01 | 0.21 ± 0.01 | 0.16 ± 0.03 | 0.27 ± 0.05 |
| Alpine | *Xerochrysum subundulatum* | 13.0 ± 1.67 | 23.9 ± 1.63 | 0.35 ± 0.03 | 0.83 ± 0.01 | 0.20 ± 0.03 | 0.16 ± 0.04 | 0.22 ± 0.03 |
| Temperate | *Acacia binervata* | 29.4 ± 0.77 | 43.4 ± 1.16 | 0.15 ± 0.01 | 0.61 ± 0.01 | 0.58 ± 0.02 | 0.04 ± 0.01 | 0.04 ± 0.01 |
| Temperate | *Acacia longifolia* | 22.1 ± 2.34 | 24.0 ± 2.44 | 0.25 ± 0.01 | 0.64 ± 0.02 | 0.49 ± 0.04 | 0.02 ± 0.01 | 0.04 ± 0.01 |
| Temperate | *Backhousia myrtifolia* | 12.7 ± 2.13 | 33.2 ± 2.45 | 0.26 ± 0.01 | 0.53 ± 0.01 | 0.50 ± 0.01 | 0.07 ± 0.02 | 0.04 ± 0.01 |
| Temperate | *Melaleuca hypericifolia* | 3.06 ± 0.26 | 11.9 ± 0.49 | 0.15 ± 0.01 | 0.55 ± 0.02 | 0.66 ± 0.03 | 0.04 ± 0.01 | 0.03 ± 0.01 |
| Temperate | *Pittosporum undulatum* | 41.8 ± 9.28 | 43.9 ± 4.86 | 0.21 ± 0.01 | 0.56 ± 0.01 | 0.56 ± 0.03 | 0.08 ± 0.02 | 0.06 ± 0.02 |
| Desert | *Acacia aneura* | 2.59 ± 0.36 | 7.05 ± 0.90 | 0.37 ± 0.06 | 0.66 ± 0.02 | 0.50 ± 0.08 | 0.27 ± 0.01 | 0.22 ± 0.02 |
| Desert | *Acacia salicina* | 9.92 ± 2.00 | 11.4 ± 2.44 | 0.35 ± 0.03 | 0.68 ± 0.01 | 0.41 ± 0.04 | 0.15 ± 0.06 | 0.20 ± 0.03 |
| Desert | *Dodonaea viscosa* | 7.92 ± 1.74 | 11.6 ± 1.40 | 0.17 ± 0.01 | 0.52 ± 0.01 | 0.72 ± 0.04 | 0.09 ± 0.03 | 0.06 ± 0.04 |
| Desert | *Eucalyptus largiflorens* | 8.91 ± 1.17 | 11.6 ± 1.48 | 0.33 ± 0.03 | 0.56 ± 0.01 | 0.53 ± 0.03 | 0.07 ± 0.01 | 0.08 ± 0.02 |
| Desert | *Flindersia maculosa* | 1.89 ± 0.14 | 7.68 ± 0.37 | 0.27 ± 0.01 | 0.57 ± 0.02 | 0.55 ± 0.02 | NA | 0.35 ± 0.00 |

LA = leaf area (cm^2^), LW = leaf width (mm), LT = leaf thickness (mm), LWC = leaf water content, LD = leaf density (g cm^-3^), *g*_sw_ = stomatal conductance to water

(mol m^-2^ s^-1^). NA indicates that measurements could not be made on these plants due to small leaf size.

**Table S4.** Principal Component Analysis (PCA) variable loadings and total explained variance for the six leaf traits.

| **Variable** | **PC1** | **PC2** | **PC3** | **PC4** | **PC5** | **PC6** |
| --- | --- | --- | --- | --- | --- | --- |
| LWC | **–0.531** | –0.116 | 0.220 | 0.568 | 0.278 | 0.506 |
| LA | 0.150 | **–0.661** | 0.111 | –0.243 | 0.671 | –0.139 |
| LW | 0.003 | **–0.697** | 0.049 | –0.062 | –0.665 | 0.257 |
| LT | **–0.472** | 0.029 | –0.595 | –0.550 | 0.102 | 0.331 |
| LD | **0.548** | 0.206 | 0.224 | –0.239 | 0.109 | 0.734 |
| *g*_sw_ | **–0.415** | 0.143 | 0.730 | –0.505 | –0.091 | –0.104 |
| Var. % | 45.2 | 30.5 | 12.0 | 7.0 | 3.9 | 1.4 |

LWC = leaf water content; LA = leaf area (cm^2^), LW = leaf width (mm), LT = leaf thickness (mm), LD = leaf density (g cm^-3^), LWC = leaf water content, *g*_sw_ = stomatal conductance to water (mol m^-2^ s^-1^). Note that the PCA was conducted on a subset of the complete dataset where 114 measurements remained after removing plants for which *g*_sw_ could not be measured due to small leaf size.

**Table S5.** Tukey’s Honest Significant Differences pairwise contrasts for Δ*T* and β among biome and treatments. Bold indicates significances at *p* < 0.05.

| **Δ*T* pairwise contrasts** | **Estimate** | **SE** | ***t-*ratio** | ***p-*value** |
| --- | --- | --- | --- | --- |
| Temperate Benign - Alpine Benign | 1.719 | 0.684 | 2.512 | 0.186 |
| Temperate Benign - Desert Benign | 2.238 | 0.498 | 4.493 | **0.005** |
| Temperate Benign - Temperate High Temperature | 1.589 | 0.371 | 4.277 | **0.001** |
| Temperate Benign - Alpine High Temperature | 3.690 | 0.684 | 5.393 | **0.001** |
| Temperate Benign - Desert High Temperature | 4.357 | 0.498 | 8.746 | **< 0.001** |
| Alpine Benign - Desert Benign | 0.519 | 0.616 | 0.844 | 0.955 |
| Alpine Benign - Temperate High Temperature | –0.130 | 0.684 | –0.190 | 1.000 |
| Alpine Benign - Alpine High Temperature | 1.971 | 0.371 | 5.306 | **< 0.001** |
| Alpine Benign - Desert High Temperature | 2.639 | 0.616 | 4.286 | **0.005** |
| Desert Benign - Temperate High Temperature | –0.649 | 0.498 | –1.303 | 0.779 |
| Desert Benign - Alpine High Temperature | 1.452 | 0.616 | 2.358 | 0.224 |
| Desert Benign - Desert High Temperature | 2.119 | 0.371 | 5.704 | **< 0.001** |
| Temperate High Temperature - Alpine High Temperature | 2.101 | 0.684 | 3.070 | 0.073 |
| Temperate High Temperature - Desert High Temperature | 2.768 | 0.498 | 5.557 | **< 0.001** |
| Alpine High Temperature - Desert High Temperature | 0.668 | 0.616 | 1.084 | 0.881 |
|  |  |  |  |  |
| **β pairwise contrasts** | **Estimate** | **SE** | ***t-*ratio** | **p-value** |
| Temperate Benign - Alpine Benign | 0.017 | 0.079 | 0.221 | 1.000 |
| Temperate Benign - Desert Benign | 0.092 | 0.064 | 1.441 | 0.703 |
| Temperate Benign - Temperate High Temperature | –0.149 | 0.047 | –3.194 | **0.025** |
| Temperate Benign - Alpine High Temperature | –0.099 | 0.079 | –1.255 | 0.800 |
| Temperate Benign - Desert High Temperature | –0.064 | 0.064 | –0.996 | 0.915 |
| Alpine Benign - Desert Benign | 0.075 | 0.067 | 1.119 | 0.868 |
| Alpine Benign - Temperate High Temperature | –0.166 | 0.079 | –2.109 | 0.368 |
| Alpine Benign - Alpine High Temperature | –0.116 | 0.047 | –2.498 | 0.138 |
| Alpine Benign - Desert High Temperature | –0.081 | 0.067 | –1.214 | 0.825 |
| Desert Benign - Temperate High Temperature | –0.241 | 0.064 | –3.765 | **0.012** |
| Desert Benign - Alpine High Temperature | –0.191 | 0.067 | –2.859 | 0.086 |
| Desert Benign - Desert High Temperature | –0.156 | 0.047 | –3.348 | **0.016** |
| Temperate High Temperature - Alpine High Temperature | 0.050 | 0.079 | 0.632 | 0.985 |
| Temperate High Temperature - Desert High Temperature | 0.085 | 0.064 | 1.328 | 0.767 |
| Alpine High Temperature - Desert High Temperature | 0.035 | 0.067 | 0.526 | 0.994 |

**References**

**Bison NN, Michaletz ST.** **2024**. Variation in leaf carbon economics, energy balance, and heat tolerance traits highlights differing timescales of adaptation and acclimation. *New Phytologist,* **242**: 1919-1931.

**Michaletz ST, Weiser MD, McDowell NG*, et al.*** **2016**. The energetic and carbon economic origins of leaf thermoregulation. *Nature Plants,* **2**: 16129.

**Michaletz ST, Weiser MD, Zhou J, Kaspari M, Helliker BR, Enquist BJ.** **2015**. Plant thermoregulation: energetics, trait–environment interactions, and carbon economics. *Trends in Ecology & Evolution,* **30**: 714-724.
